# Mechanical Tests Applied to Dental Cements

**Published:** 1899-11

**Authors:** Virgil C. Pond

**Affiliations:** Boston, Mass.


### MECHANICAL TESTS APPLIED TO DENTAL CEMENTS.[1]

BY VIRGIL C. POND, B.P., D.M.D., BOSTON, MASS.

In these days of scientific accuracy, when everything is tested mechanically and chemically, when the thread is broken and the finished cloth torn apart; when iron is broken and tested at every step from the pig to the finished product; when cements used in construction are mixed, set, and broken to a scale in pounds before being used; when all workers seek to learn the value of the material they are to use, it seemed to me that something might be learned about our cement filling by the use of a testing machine.

Testing machines are now made for a great variety of purposes, and are very extensively used. Among them are many forms for testing building cements, which are used by architects. A good and very interesting collection of testing machines may be seen at the Massachusetts Institute of Technology, Boston. Riehlé Bros., of Philadelphia, manufacturers of testing machines, constructed a very ingenious machine for me, designed to crush or break small objects, and during the past five years I have tested quite a variety of materials with it.

With this machine there are a number of dies in which fillings of any kind can be made; the fillings are removed from the dies, placed in the testing machine, between two steel plates, which close practically parallel to each other, yet with a slight play upon rods and pins so that the filling to be tested is evenly grasped. The power is applied with a wheel and screw, and measured on a self-registering scale, which gives the exact crushing- or breaking-point of the filling in pounds.

Not all the things possible or thought of have been tried, and some of the tests have not been worked out, perhaps, as far as they should have been, but definite results were arrived at in some cases, and much, to me, useful knowledge gained.

The first set of experiments were directed towards finding out how to mix a cement filling. Different cements were taken and mixed in three ways, first to a creamy consistence, or rather thicker,

---

[1] A paper read before the Alumni of the Harvard Dental School, on Alumni Day, June 26, 1899.

as it is practically impossible to fill a die with cement of that consistence; second, to a fairly stiff mass, which could be taken between the fingers; and, third, to a point where the greatest possible amount of powder was incorporated with the liquid, in fact, mixed according to the directions given by many manufacturers, using a strong, thick, wide spatula.

These mixtures were packed in the dies, allowed to harden in the same length of time, and then broken with the machine.

The experiments were repeated many times to avoid errors, and the figures given are the average results of all the tests made.

The difficulty of making a perfect filling in die, under the most favorable conditions, so often alluded to by writers, was fully realized; and it is my belief that many cement fillings fail because they are not properly packed, the cavity is not thoroughly filled, and I think you will agree with me if you will fill some dies with cement and examine the fillings under a magnifying glass.

The average with fourteen of the cements in common use was as follows: Taking the breaking-point of the mixture with the greatest amount of powder incorporated at 100, the breaking-point of the medium mixture was 80.8 per cent., and the thin mixture about 51 per cent. I say about 51 per cent., because with several of the kind the thin mixtures had not set sufficiently in the time allowed (fifteen hours) to have any strength, and they had to be omitted from the calculation. It seems to be a law that the more powder worked into the fluid the stronger the resulting mass. It did not matter whether the cement was a strong or a weak one, no exception to the rule was found.

The time of setting made no difference, as the same results were obtained at all times of trial, from one hour to several days; they were all stronger after the longer periods of time, but the relative strength was the same. The time generally used (fifteen hours) was taken for convenience simply, as it was found that the hardest or driest mixtures had thoroughly set in that time, and some fixed time was needed in order to compare different makes of cement. The slow setting of the soft or thin mixtures will be referred to again.

In the above tests it was noticed that different colored powder, of the same make, out of the same box, mixed with the same liquid, varied considerably in strength.

These were submitted to an expert professional chemist, who reported that the different colored powders, where there was difference in strength of the filling noticed, were of different composition. When they were colored by the addition of some substance before fusing, or by a different degree of heat in fusing, the different colors were of about the same strength.

If you take any powder and add a small quantity of the coloring-matter, such as is furnished by some makers, there is no appreciable loss of strength, but if the coloring material is added freely or to excess, the mass is materially weakened.

We frequently hear that some one has analyzed and found arsenic in the cements, but in none of those analyzed did the chemist find any.

His analysis shows that the powders may be roughly divided into two classes: First, those which are composed of pure or practically pure oxide of zinc; second, those which contain other substances, such as oxide of tin, aluminum, silicate, and calcium.

These substances are used very largely by some makers, and form what might be called a concrete, in which they are cemented by the oxide of zinc.

The first class I have always found the strongest; some of the second class are absolutely worthless, and they all give very uneven or irregular results, owing, apparently, to the uneven mixing of the ingredients. Almost all the liquids are the glacial phosphoric acid, and most of them contain a trace of iron.

Probably all of you have had mixtures of cement harden or set very quickly; perhaps while you were manipulating them they suddenly became hard. Such mixtures were found to have no regular breaking-point, and while never more than 50 per cent. of the normal strength of the cement, they were frequently as low as 25 per cent. Chemists say this action is due to the deterioration or change of the liquid. Glacial phosphoric acid ($HPO_3$) will slowly change to common phosphoric acid ($H_3PO_4$).

To find the effect of pressure, two dies were filled equally carefully from the same mixture, and one was immediately subjected to pressure, while the other was not. After allowing to set in this manner (one under pressure and one not) they were removed from the dies and broken. It was found that the one subjected to pressure was always the stronger, the average of all tests showing 11 per cent. increased strength. Whether this increase of strength is produced by the pressure or is due simply to a more perfect packing of the material, it shows the advantage of applying all the pressure possible to a cement filling.

Tests were next made with a view to finding the time required for a cement to set.

Thin mixtures were found to be very slow in setting, and after twenty-four hours several makes flattened out like putty under a few pounds of pressure. This seems important in selecting a cement for use in crown- and bridge-work, and would suggest that you try the cement you are now using by making a filling or pellet of a thin mixture and trying it from time to time to see if it ever becomes hard and strong.

Each kind or make requires a different time, for all those tested by me required much more time than was anticipated.

Taking a hard or dry mixture (one with a large amount of powder) of one of our quickest-setting cements, it was found that fillings made in the dies would flatten out under slight but increasing pressure up to about twenty-five minutes. Between twenty-five and thirty minutes a great change took place, and at thirty minutes they broke under a pressure somewhat approaching that of their greatest strength.

The fillings cut and appeared fairly hard before they would break, but after the breaking-point was reached they were noticeably more difficult to cut.

Experiments were made to determine the adhesive force of cements. Taking as the definition of adhesion the molecular attraction exerted between the surfaces of bodies in contact, and of cohesion the force by which the molecules of the same material are bound together, it was found, with smooth bone or ivory, which was used, that the cohesion was greater than the adhesion; the cement would break off from the bone or ivory. The thin mixtures were more adhesive than the very thick ones; still, the cohesion was greater than the adhesion. When the surfaces of the bone or ivory were considerably roughened, but with no undercuts, the adhesion was greater than the cohesion, with both thick and thin mixtures, but the thick required the most force to break, as in this case it was the cement which broke.

I am unable to give the results in figures, as it was impossible to prepare and roughen the pieces of bone and ivory just alike for each test.

To learn the value of some of the preparations used to protect cement fillings while setting, several dies were filled from the same mixture, and the fillings removed as soon as possible, just as soon as they would retain their shape. The time was from two to four minutes. One filling was allowed to set without treatment; one was covered with vaseline; one was covered with some kind of varnish, and one with some one of the preparations which require heat for their application, such as the sticks furnished with some of the cements, and apparently composed of wax, paraffine, etc., and White's temporary stopping, which is recommended for this purpose.

The results of many experiments were as follows: Taking the breaking-point of the filling which was not treated, had nothing put in it, as 100, the one covered with vaseline was 76 per cent. as strong; the one covered with varnish was 71.2 per cent. as strong; the one covered with heated preparation was 67.6 per cent. as strong. All the preparations had a distinctly weakening effect. The experiment next made was exactly as above, except that the fillings were dropped into saliva as soon as treated, and allowed to remain there until set.

One more filling was made for this test, and two were left untreated, one of which was allowed to set out of the saliva, and one was put into the saliva with the treated filling. Taking the breaking-point of the filling which was left out of the saliva, one of the untreated ones, at 100, the other untreated one, which set in saliva, was 76 per cent. as strong; the vaseline-covered one, 69.7 per cent.; the varnish-covered one, 62 per cent.; the heated preparation, 49 per cent. The untreated fillings, although they set in the saliva, were stronger than the protected ones. The same experiments were made, allowing a longer period of time before treating and putting them into the saliva, but the results were relatively the same, although all were stronger. After about thirty minutes, or the time when the cement sets, the saliva has much less effect upon the fillings, and the untreated or unprotected filling, placed in it, approaches in strength its mate, which hardens in the air. The figures at thirty minutes were, 100 + 82 per cent.

With the varnishes the loss of strength was due to the ether and alcohol, in which the gums are dissolved. With the hot applications it seems to be due to the heat. I find that, in order to cover the filling sufficiently to protect it from moisture, the preparation must be thoroughly heated, in fact, practically melted. If this is not done, you can see under a magnifying glass that it simply adheres to the filling in places, but does not cover it, and affords but little protection against moisture.

A filling placed in a sand-bath, heated to a temperature sufficient to thoroughly soften one of these preparations, and left there a few seconds, will show the same loss of strength.

According to the results of these tests, it is my opinion that, first, in mixing any cement, as much powder as possible, consistent with good working qualities, should be incorporated; secondly, if coloring material is used, it should not be used to excess; thirdly, if the mixture acts poorly, sets immediately, or before it can be properly packed, it should not be used; fourthly, all pressure possible should be applied to the filling, using a matrix, wherever necessary, to get pressure; fifthly, the cement should be placed in a perfectly dry cavity, and kept thoroughly dry until packed (you will get fair results with most cements if they are kept dry two or three minutes, very good results if kept dry from eight to ten minutes, and the best possible results when kept dry from twenty-five to thirty minutes); sixthly, none of the so-called protecting coatings I have tried should be used.

These experiments have been duplicated in the mouth as far as possible, and, to the best of my judgment, the results obtained from the testing machine have been confirmed in practice.

No doubt many of you have tried these experiments, although, perhaps, in a different way, and the discussion will show whether our results agree.

If the testing machine proves reliable, it will be much better and more simple than any chemical tests, for with the composition of a cement known, its strength and durability is yet to be determined, and every box must be analyzed, to know that the manufacturer had not changed its composition without altering the label. Each box *can* be tested by a machine, and every make compared with little trouble.

In closing, I will quote from Dr. W. B. Ames, who has made a study of cements from what may be called the chemical side. He says, "The prime cause of the destruction of a good phosphate filling in the mouth is friction."

